# Human Monkeypox without Viral Prodrome or Sexual Exposure, California, USA, 2022

**DOI:** 10.3201/eid2810.221191

**Published:** 2022-10

**Authors:** Abraar Karan, Ashley R. Styczynski, ChunHong Huang, Malaya K. Sahoo, Krithika Srinivasan, Benjamin A. Pinsky, Jorge L. Salinas

**Affiliations:** Stanford University School of Medicine, Stanford, California, USA

**Keywords:** monkeypox, viruses, orthopoxvirus, epidemic, United States

## Abstract

We report human monkeypox in a man who returned to the United States from the United Kingdom and reported no sexual contact. He had vesicular and pustular skin lesions but no anogenital involvement. The potential modes of transmission may have implications for the risk of spread and for epidemic control.

The 2022 multicountry monkeypox outbreak has been linked primarily to intimate contact among men who have sex with men (*1*,*2*). We describe a case of monkeypox in a traveler who returned from the United Kingdom to the United States who did not report recent sexual contact. 

A man in his 20s sought care at an emergency department in Stanford, California, USA, on day 7 of an asynchronous, diffuse vesicular rash following travel to the United Kingdom. The first lesion appeared ≈14 days after he attended a large, crowded outdoor event at which he had close contact with others, including close dancing, for a few hours. He said that many attendees were in sleeveless tops and shorts. He wore pants and a short-sleeved top. He did not notice any skin lesions on anyone present, nor did he notice anyone who seemed sick. He shared an e-cigarette with a woman that he met while there. The event was not a rave and was not attended specifically or mostly by persons identifying as gay or bisexual. He attended other similar outdoor events over 4 days. He reported consuming alcohol but no other drug use at these events. He did not wear a mask at these events. He had contact with domestic dogs that he petted. 

He took 2 flights to return to the United States; masks were worn on 1 flight. He identifies as bisexual but reported no recent sexual contacts during his travels or in the preceding 3 months. He reported no close indoor activities, although he traveled on crowded public trains. He reported no close contacts since his return. He lives with 1 roommate who did not manifest any symptoms. He had a history of syphilis treated 3 months earlier and was taking HIV preexposure prophylaxis. He denied preceding fevers, chills, headache, lymph node swelling, cough, fatigue, or anorectal pain.

We noted multiple nondraining skin lesions at different stages of appearance, including a centrally umbilicated vesicle on his left palm, a crusting flat lesion on his lip, and pustules on his right and left knuckles and on his lateral torso and back. He had no penile, testicular, or anal lesions and no cervical, axillary, or inguinal lymphadenopathy (Appendix).

Results of complete blood count and basic metabolic panel results were unremarkable. Results of rapid HIV-1 antibody/antigen test was negative, as was urine testing for *N. gonorrhoeae* and *C. trachomatis*. Rapid plasma regain test results were positive (titer of 1:1). The palmar vesicle was unroofed; a swab of the expressed clear fluid tested positive for nonvariola orthopoxvirus DNA by quantitative PCR (qPCR) and was confirmed as monkeypox virus DNA by qPCR specific for clade 2/3 (West Africa) monkeypox (Table; Appendix). A nasopharyngeal swab specimen that tested negative for SARS-CoV-2 was positive for monkeypox virus DNA using this 2-step testing algorithm. We did not prescribe specific monkeypox treatment because the patient did not have complications or risk factors for severe disease.

**Table:** Monkeypox virus DNA levels in clinical specimens from a man with monkeypox. California, USA, 2022*

Specimen type	Days since symptom onset	Viral copies/mL	log_10_ copies/mL	Ct value
Lesion swab	7	65,647,690	7.82	12.7
Nasopharyngeal swab	7	1,200	3.08	27.6
Saliva	10	3,030	3.48	26.3
Rectal swab	10	Detected, unable to quantitate	NA	30.2
Conjunctival swab	10	Detected, unable to quantitate	NA	32.6
Oropharyngeal swab	10	Not detected	NA	NA
Semen	10	Not detected	NA	NA

*The concentrations of the lesion, and self-collected rectal and conjunctival swab specimens are expressed in copies/mL phosphate-buffered saline (PBS). These samples, as well as the self-collected oropharyngeal swab, were collected dry and rehydrated with 1 mL PBS. The nasopharyngeal swab was healthcare worker–collected in 3 mL viral transport media. Cycle threshold values and estimated concentrations are based on the results from the non-variola orthopoxvirus quantitative PCR. All samples with values in copies/mL or reported as detected, unable to quantitate, were positive by both the non-variola orthopoxvirus qPCR and the clade 2/3 monkeypox qPCR. Ct, cycle threshold; NA, not applicable.

We performed follow-up monkeypox virus testing with patient consent 3 days after initial evaluation (day 10 after symptom onset) to clarify viral shedding. We detected virus DNA in a saliva sample, as well as from patient-collected conjunctival and rectal swabs using both the non-variola orthopoxvirus and clade 2/3 monkeypox virus qPCRs. Lesions resolved by day 26 after symptom onset.

This patient tested positive for monkeypox virus DNA from several nonlesion samples. The nasopharyngeal and saliva findings are noteworthy because the patient did not report respiratory symptoms. In addition, the detectable viral DNA in the rectal swab specimen in the absence of visible anal lesions or pain indicates a potential for sustained sexual transmission, although the viral DNA levels were low; contamination during self-collection cannot be ruled out. We were unable to assess whether internal rectal lesions were present. 

This case highlighted the distinctiveness of clinical manifestations as they indicated potential routes of transmission during the 2022 multicountry outbreak of monkeypox. This patient did not report recent sexual contact, did not have evidence of genital lesions or inguinal lymphadenopathy (*3*), and did not report a viral prodrome. His primary risk factor was close, nonsexual contact with numerous unknown persons at a crowded outdoor event. His case highlights the potential for spread at such gatherings, which may have implications for epidemic control. The lack of both sexual exposure and anogenital involvement indicates that mode of transmission may be associated with clinical symptoms; fomites (hotel bedding and sheets, high-touch areas in public settings) may be alternative modes of transmission. Overall, the viral inoculum required for all possible modes of transmission remains an area of active investigation.

This case also demonstrates the importance of local monkeypox virus testing, rather than centralized testing in public health or commercial reference laboratories. Local testing enabled diagnosis in <12 hours and immediate notification to local and state public health authorities for isolation and contact tracing. 

Appendix Additional information about monkeypox in a patient without viral prodrome or sexual exposure, California, USA.
