# Supplementary material for: Human Monkeypox without Viral Prodrome or Sexual Exposure, California, USA, 2022
Source: Emerg Infect Dis. 2022 Oct;28(10):2121–3. doi: 10.3201/eid2810.221191 (PMC9514362; doi:10.3201/eid2810.221191)
Supplement: Appendix — Additional information about monkeypox in a patient without viral prodrome or sexual exposure, California, USA. [file 22-1191-Techapp-s1.pdf]

Article DOI: https://doi.org/10.3201/eid2810.221191

Human Monkeypox without Viral Prodrome or Sexual Exposure, California, USA, 2022

Appendix

Methods

Lesion, rectal, conjunctival, and oropharyngeal swab samples were placed in conical tubes without transport media (dry) and rehydrated in 1mL phosphate buffered saline at the Stanford Clinical Virology Laboratory. The nasopharyngeal swab was collected in 3 mL viral transport media. Saliva and semen were collected in sterile containers. Total nucleic acids were purified from 400 µL of each sample using the EZ1 Virus Mini Kit version 2.0 on the EZ1 Advanced XL instrument (QIAGEN, https://www.qiagen.com) and eluted in 60 uL of buffer AVE.

Testing for monkeypox virus DNA was performed using 2 laboratory-developed quantitative PCR (qPCR) assays modified from the published work of the US Centers for Disease Control and Prevention (*1*,*2*). The first screening qPCR targets viral DNA polymerase sequences conserved throughout the non-variola orthopoxviruses, including monkeypox. The second confirmatory qPCR targets viral TNF receptor sequences specific for clade 2/3 (West Africa) monkeypox viruses. All samples reported in this manuscript as positive for monkeypox virus DNA were detected by both qPCRs.

These assays were performed using the Luna Universal Probe qPCR Master Mix (New England Biolabs, https://www.neb.com) on the Rotor-Gene Q instrument (QIAGEN). Each reaction was performed using 10 µL of eluate, with a final reaction volume of 25 µL. Primers and FAM-labeled probes were obtained from Integrated DNA Technologies and were added at final concentrations of 300 nM and 100 nM, respectively. As internal control (IC), primers and HEX-labeled probes targeting the human β-globin gene were added in multiplex at final concentrations of 100 nM and 50 nM, respectively (*3*). Cycling conditions were as follows: hold at 94°C for 2 minutes, followed by 45 cycles of 94°C for 15 seconds, 60°C for 30 seconds, and

72°C for 10 seconds. Detection was performed in the green (non-variola orthopoxvirus or clade 2/3 monkeypox) and yellow (IC:β-globin) channels; the threshold was set at 0.05 for both channels. To report a sample as negative for virus DNA, the IC must be amplified at a cycle threshold (Ct) <35 cycles. This ensures adequate specimen collection and nucleic acid extraction, as well as the absence of PCR inhibitors.

The non-variola orthopoxvirus qPCR was calibrated using quantitated double stranded DNA containing the assay target sequence (gBlock; Integrated DNA Technologies, https://www.idtdna.com). The 95% lower limit of detection (LLOD) was determined using serial dilutions of purified nucleic acids from a quantitated high-positive sample and calculated using probit regression. The non-variola orthopoxvirus qPCR has a 95% LLOD of 81 copies/mL (95% CI 59–332 copies/mL). Based on further precision experiments, the lower limit of quantitation (LLOQ) was determined to be 500 copies/mL.

References

1. Li Y, Olson VA, Laue T, Laker MT, Damon IK. Detection of monkeypox virus with real-time PCR assays. J Clin Virol. 2006;36:194–203. PubMed https://doi.org/10.1016/j.jcv.2006.03.012

2. Li Y, Zhao H, Wilkins K, Hughes C, Damon IK. Real-time PCR assays for the specific detection of monkeypox virus West African and Congo Basin strain DNA. J Virol Methods. 2010;169:223–7. PubMed https://doi.org/10.1016/j.jviromet.2010.07.012

3. Mills AM, Guo FP, Copland AP, Pai RK, Pinsky BA. A comparison of CMV detection in gastrointestinal mucosal biopsies using immunohistochemistry and PCR performed on formalin-fixed, paraffin-embedded tissue. Am J Surg Pathol. 2013;37:995–1000. PubMed https://doi.org/10.1097/PAS.0b013e31827fcc33
